# Editorial: COVID-19 and diabetes: Current findings and future perspectives

**DOI:** 10.3389/fendo.2024.1421721

**Published:** 2024-05-09

**Authors:** Pranav Kumar Prabhakar

**Affiliations:** Research and Development Cell, Lovely Professional University, Phagwara Punjab, India

**Keywords:** diabetes, COVID - 19, complication, SARS-CoV2, cardiovascular

## Introduction

COVID-19 and diabetes represent a complex intersection in the realm of public health, with profound implications for individuals’ health outcomes and healthcare systems worldwide. The COVID-19 pandemic, caused by the novel coronavirus SARS-CoV-2, has rapidly spread across the globe since its emergence in late 2019, resulting in millions of infections and deaths (1). Individuals with diabetes have been identified as a particularly vulnerable population, facing an increased risk of severe outcomes from COVID-19. Conversely, COVID-19 infection can exacerbate glycemic control and increase the risk of diabetic complications, highlighting the bidirectional relationship between these two conditions.

Diabetes is a chronic metabolic disorder characterized by elevated blood sugar levels due to insulin deficiency or resistance. It encompasses several subtypes, including type 1 diabetes, type 2 diabetes, and gestational diabetes, each with its own etiology and management considerations. Diabetes affects millions of people worldwide and is associated with a range of complications, including cardiovascular disease, kidney failure, neuropathy, and retinopathy (2).

Despite these challenges, the COVID-19 pandemic has also catalyzed innovation and adaptation in diabetes care delivery. Telemedicine, remote monitoring, and digital health technologies have emerged as valuable tools for delivering diabetes care and education remotely, enhancing access and convenience for patients. Healthcare providers have embraced virtual consultations, telehealth platforms, and mobile applications to maintain continuity of care and support patients in managing their diabetes during the pandemic (3). These digital health solutions offer opportunities to reach underserved populations, improve patient engagement, and optimize diabetes outcomes in the era of COVID-19 and beyond. This Research Topic aimed at the relationship and coexistence of diabetes and COVID-19. The Research Topic currently includes 16 papers which contain 2 case studies, 2 mini reviews, and 12 original research articles on the various topics.

## Interactions between COVID-19 infection and diabetes

The relationship between COVID-19 and diabetes is multifaceted and multifactorial. Individuals with diabetes are more likely to experience severe outcomes from COVID-19, including hospitalization, admission to intensive care units, and death. Several factors contribute to this heightened risk, including impaired immune function, underlying comorbidities, and physiological changes associated with diabetes. Moreover, individuals with diabetes often have other risk factors for severe COVID-19 outcomes, such as obesity, hypertension, and cardiovascular disease, further increasing their vulnerability. Conversely, COVID-19 infection can exacerbate glycemic control and increase the risk of diabetic complications (4). The inflammatory response triggered by COVID-19 can lead to insulin resistance and hyperglycemia, particularly in individuals with pre-existing diabetes. Furthermore, the stress of illness, changes in diet and physical activity, and disruptions to routine diabetes care can all contribute to worsening glycemic control during COVID-19 infection. Yuanyuan et al. analyzed the web of science database for the comprehensive analysis of current publications related to diabetes mellitus (DM) research during the COVID-19 epidemic reveals a growing body of literature addressing various aspects of this intersection.

The COVID-19 pandemic, caused by SARS-CoV-2, has profoundly affected global health and economy since 2020. The virus binds to ACE2 receptors, found in various organs including endocrine glands, impacting multiple endocrine systems. COVID-19 promotes obesity through lifestyle changes, exacerbating diabetes risk. Additionally, it directly affects pancreatic function, worsening type 1 or type 2 diabetes. High adiposity and chronic hyperglycemia increase COVID-19 susceptibility and severity. Bidirectional interactions exist between COVID-19 and diabetes, influencing each other’s progression. Healthcare systems have adapted services to manage diabetes amidst the pandemic’s challenges. Overall, COVID-19 and diabetes share complex interactions, necessitating tailored healthcare responses. Wolińska et al. discussed the role of various environmental factors that lead to obesity either before COVID-19 or after COVID-19. The rapid rise in overweight and obesity over recent decades has been influenced by various factors, including environmental and novel elements emerging during the COVID-19 pandemic. Lockdown measures during the pandemic led to increased BMI in many countries, driven by reduced physical activity, increased screen time and sleep duration, and elevated consumption of processed foods. Environmental factors such as policy issues, socioeconomic status, lifestyle choices, and neighborhood conditions also contribute to obesity trends. Air pollution’s role in obesity remains debated. However, the pandemic’s impact extends beyond weight gain, affecting individuals with diabetes disproportionately.

## Challenges during hospitalization or post covid-19

In this nationwide retrospective investigation, Kania et al. examined the association between diabetes and in-hospital mortality among COVID-19 patients. Conducted in Poland since the pandemic’s onset in 2019, the study revealed diabetes as a significant factor linked to increased hospitalization rates and higher risk of in-hospital mortality, even after adjusting for various factors like age, sex, and comorbidities such as chronic kidney disease and heart conditions. The study also noted variations in relative risk across different age groups and genders, with heightened risks observed in males and patients in their sixties. The research underscores the importance of recognizing diabetes as a crucial risk factor in COVID-19 prognosis, offering valuable insights for healthcare providers.

Meanwhile, Gorchane et al. and Bukara-Radujkovic et al. investigated the impact of the COVID-19 pandemic on new-onset diabetic ketoacidosis (DKA) in Africa, an area with limited prior research on this topic. Their analysis compared DKA incidence trends before and during the pandemic, highlighting an increase in DKA cases alongside rises in both type 1 and type 2 diabetes. These findings suggest that the pandemic may have contributed to the observed uptick in DKA cases, emphasizing the need for further research and attention to COVID-19’s effects on diabetes-related complications in Africa (9, 10).

## Case study

A case of young pregnant Chinese woman developed sudden hyperglycemia and ketoacidosis in her last trimester, following mild SARS-CoV-2 infection were studies. Despite near-normal glycohemoglobin levels, low C peptide levels indicated severe insulin deficiency, leading to a diagnosis of fulminant type 1 diabetes (FT1D). Insulin therapy swiftly improved ketoacidosis and hyperglycemia, but β cell function remained impaired. The patient transitioned to insulin pump therapy post-discharge, with favorable glucose control at the first follow-up. This case underscores the potential for FT1D onset following SARS-CoV-2 infection and the importance of prompt recognition and management during the COVID-19 pandemic (11). Another case study of a 16-year-old boy developed symptoms of polyuria, polydipsia, and weight loss after receiving the BNT162b2 Comirnaty COVID-19 vaccine, worsening after the second dose has been performed. Diagnostic tests revealed central diabetes insipidus due to neuroinfundibulohypophysitis. Treatment with Desmopressin alleviated symptoms, with ongoing follow-up. This case highlights the need for vigilance in recognizing and reporting potential adverse effects of COVID-19 vaccines, including rare conditions like hypophysitis. Further research is required to determine any causal link between COVID-19 vaccination and the development of central diabetes insipidus (12).

## Future prospective

A large number of articles were focused on the prediction of various aspects of post covid complications. This study done by Byeon employed machine learning techniques to identify major risk factors for depression in community diabetic patients and developed predictive models for high-risk group identification. Analyzing 26,829 adults diagnosed with diabetes, it found a 22.4% prevalence of depression. Utilizing CatBoost, the top nine influential factors included gender, smoking status, COVID-19-related changes in drinking and smoking, subjective health, economic concerns, sleep alterations, economic activity, and social support. Early identification of high-risk individuals is crucial for implementing personalized psychological support at the primary medical level, enhancing mental health outcomes for diabetic patients (13).

Shoaib et al.‘s study underscores the complex interplay between COVID-19 and diabetes, recognizing heightened vulnerability and potential post-complications for diabetic individuals. Additionally, it suggests a potential association between cough medicine containing steroids and an increased risk of developing diabetes. The study utilized deep-learning models on chest x-ray images sourced from publicly available datasets, validated by a certified radiologist, to aid diagnosis (14). Another study by Ahmad et al. explored a deep transfer learning approach for COVID-19 detection, achieving a high accuracy of 99.11% with the CIDICXR-Net50 model. This study also investigated the relationship between COVID-19 and diabetes, aiming to enhance diabetes prediction through advanced machine learning techniques. Initial assessment favored the Support Vector Machines (SVM) classifier with 76.62% accuracy (15). Advanced feature engineering revealed hidden patterns, particularly in Glucose levels. Correlation analyses highlighted significant associations, and integrating Decision Trees, Gradient Boosting, and SVM in an ensemble model improved accuracy to 93.2%. This research offers a robust framework for diabetes prediction, crucial for early diagnosis, personalized treatment, and preventive care, addressing global health challenges and enhancing life expectancy (16).

Future prospects regarding COVID-19 and diabetes are multifaceted, encompassing various aspects of research, treatment, preventive measures, healthcare infrastructure, public health policies, and education. Ongoing research endeavors aim to elucidate the complex interaction between COVID-19 and diabetes, seeking to understand why individuals with diabetes face a heightened risk of severe outcomes from the virus. This research is crucial for developing targeted treatments to mitigate complications and improve outcomes for diabetic patients infected with COVID-19. In addition to research, future strategies are likely to prioritize preventive measures tailored to individuals with diabetes. This may include vaccination campaigns aimed at diabetic populations, lifestyle interventions to manage diabetes effectively, and improved diabetes management protocols to reduce the risk of severe COVID-19 outcomes. Furthermore, the pandemic has underscored the importance of robust healthcare infrastructure, particularly for managing chronic conditions like diabetes during public health crises. Investments in telemedicine, remote monitoring technologies, and integrated care models are anticipated to enhance the delivery of healthcare services and improve outcomes for diabetic patients during future outbreaks. Moreover, public health policies may be developed to address the intersection of COVID-19 and diabetes. These policies could involve prioritizing vaccination for diabetic individuals, ensuring equitable access to healthcare services, and implementing measures to reduce the risk of COVID-19 transmission in vulnerable populations. Concurrently, education and awareness efforts will likely intensify, emphasizing the increased risk of COVID-19 complications among individuals with diabetes. Such campaigns may promote vaccination uptake, adherence to preventive measures such as mask-wearing and social distancing, and regular monitoring of blood glucose levels to manage diabetes effectively in the context of the pandemic.

In conclusion, the intersection of COVID-19 and diabetes presents complex challenges and opportunities for healthcare systems, policymakers, and individuals alike. By prioritizing vaccination efforts, optimizing diabetes management, strengthening healthcare infrastructure, investing in research and innovation, and promoting health equity, we can mitigate the impact of COVID-19 on individuals with diabetes and safeguard their health and well-being in the face of future pandemics.

## Author contributions

PP: Writing – review & editing, Writing – original draft.
